# Cost-effectiveness of alectinib compared to crizotinib for the treatment of first-line ALK+ advanced non-small-cell lung cancer in France

**DOI:** 10.1371/journal.pone.0226196

**Published:** 2020-01-16

**Authors:** Marine Sivignon, Rémi Monnier, Bertrand Tehard, Stéphane Roze

**Affiliations:** 1 Heva Heor, Lyon, France; 2 Roche, Boulogne-Billancourt, France; University of South Carolina College of Pharmacy, UNITED STATES

## Abstract

The aim of the study is to evaluate the cost-effectiveness of alectinib for first-line treatment of ALK+ advanced non-small-cell lung cancer compared to crizotinib in the French setting. This study used a partitioned survival model, with three discrete health states (progression-free survival, post-progression survival and death). Survival probabilities were derived from a randomised Phase III clinical trial comparing alectinib to crizotinib (ALEX). Beyond the length of the trial (18 months), the efficacy of both treatments was considered equivalent. Occurrence of adverse events or brain metastases were considered as inter-current events. Utilities (and disutilities for intercurrent adverse events) derived from the EQ-5D were applied. Costs were attributed using standard French national public health tariffs. Projected mean overall survival was 4.62 years for alectinib and 4.18 years for crizotinib. Projected mean progression-free survival was 30.30 months for alectinib and 16.13 months for crizotinib. The total number of quality-adjusted life years projected was 3.40 for alectinib and 2.84 for crizotinib. The projected total cost of treatment over the lifetime of the model was € 246,022 for alectinib and € 195,486 for crizotinib. This extra cost was principally attributable to treatment acquisition costs and management before progression. Alectinib was associated with lower costs related to brain metastases and to management post-progression. The incremental cost per life year gained was 115,334 €/year and the incremental cost-effectiveness ratio was 90,232 €/QALY. First-line treatment of ALK+ NSCLC with alectinib provides superior clinical outcomes to crizotinib and is cost-effective in the French context.

## Introduction

Lung cancer is the leading cause of cancer-related death in Europe [1], with an estimated five-year survival rate of ≤20% [2]. In France, lung cancer is the second most common cancer in men and the third most common in women, with an estimated 45,000 incident cases and 30,500 lung cancer-related deaths in 2015 [2]. Prognosis is generally poor regardless of the histological type of lung cancer and response to chemotherapy has historically been limited.

However, the discovery of molecular fingerprints in certain forms of lung cancer has led to the development of targeted therapies which offer the promise of greater effectiveness in those patients who develop cancers bearing these fingerprints [3]. In particular, inhibitors of the epidermal growth factor receptor (EGFR), such as gefitinib and erlotinib, or anaplastic lymphoma kinase (ALK), such as crizotinib and alectinib, have been developed. Mutations in the Erb-1 gene encoding EGFR are present in 10–20% of patients developing squamous cell lung cancer and rearrangements of the ALK gene are present in around 3.2% of patients developing non-small cell lung cancer (NSCLC) [3, 4].

Crizotinib, the first ALK inhibitor to be introduced for the treatment of lung cancer in 2011, has rapidly become the gold standard for this type of cancer [5, 6]. However, its long-term effectiveness is compromised by the development of resistance and progression of central nervous system (CNS) disease [7]. Alectinib is an orally-administered inhibitor of ALK which has been demonstrated to be effective in the treatment of ALK+ NSCLC [8]. Since this molecule is lipophilic and is not a substrate for p-glycoprotein, it penetrates the CNS effectively and can prevent the growth or occurrence of brain metastases [9]. This is of particular importance in lung cancer, where the brain is the most frequent site of metastasis [10] and where the development of brain metastases will aggravate the burden of lung cancer and compromise survival [11]. This is an important differentiating factor for alectinib compared with other treatment options for ALK+ NSCLC [12]. First-line treatment for NSCLC with alectinib has been demonstrated to be associated with longer progression-free survival and lower toxicity than crizotinib and to show activity against CNS metastatic disease [13, 14].

Little data is currently available on the costs and utilities associated with the use of alectinib compared to alternative treatments [13]. In addition, each country has different systems for provision and funding of healthcare in oncology, which necessitate evaluation of the relative benefits of different treatment strategies to be performed on a country-specific basis. In this context, the French health authorities have requested a cost-effectiveness evaluation of the treatment of first-line ALK+ advanced non-small-cell lung cancer with alectinib compared to crizotinib in the French setting.

## Methods and materials

This was a cost-effectiveness analysis of the use of alectinib in the treatment of ALK+ NSCLC in the French context compared with a reference treatment with crizotinib. The analysis was requested by the Economic and Public Health Evaluation Committee (*Commission d’Évaluation Économique et de Santé Publique*; CEESP) of the French Health authorities from the marketing authorisation holder (MAH) of alectinib (Roche) as part of the licensing process for alectinib in France. The analysis protocol was conceived by the MAH and submitted to the CEEPS for comments before implementation.81-88

### Study design

Outcomes and costs were simulated in a partitioned survival model. The analysis evaluated direct production costs and was performed from a collective perspective, taking into account costs borne by the national health insurance (NHI), costs borne by complementary private health insurance and costs borne by the patient. The time frame of the analysis was ten years.

### Description of the model

The model is a partitioned survival model, consisting of three discrete health states, namely progression-free survival (PFS), post-progression survival (PPS) and death. Transition from state to state (from PFS to PPS or death and from PPS to death) was unidirectional and irreversible. At the start of the model, all patients were in the PFS health state. The time in each health state was estimated using partition survival methods as the area under the survival curves. The cycle length was one week and the time horizon of the model was ten years, based on the assumption that given the mortality rate among these patients a ten-year period would thus be able to determine comprehensively all benefits and costs related to the two treatments compared. A discount rate of 4% was applied and adjustments were made for half-cycles.

The incidence of adverse events (AEs) and occurrence of brain metastases (BM) were considered as inter-current events and integrated into the model for determination of costs. Costs and disutilities were applied for AEs and costs only for BM.

### Model inputs

#### Analysis population

The analysis population modelled corresponded to patients with ALK+ NSCLC requiring first-line treatment included in the Phase III ALEX randomised clinical trial [13]. The profile of the patients enrolled in this trial is considered to be comparable to all patients with ALK+ NSCLC in France [15]. Patients enter the model at the age of 55 years.

#### Treatment pathways

Patents in the PPS state could be switched to a second- or third- line treatment. The mean duration of first-line alectinib was set at 32.6 months and the mean duration of first-line crizotinib at 16.7 months, these being the values observed in the ALEX trial [13]. For patients who progressed, treatment was considered to be changed. In the baseline scenario, the second-line treatment was in each case another ALK inhibitor. For patients treated with alectinib in first line, second-line treatment was ceritinib in all cases. For those treated with crizotinib in first line, second-line treatment was alectinib in 80% of cases and ceritinib in 20%. The mean duration of second-line treatment was set at 50.1 weeks, irrespective of the nature of the treatment. This value corresponds to the mean values of the duration of PFS reported in clinical trials evaluating the use of alectinib [16], crizotinib [17] and ceritinib [17]. Third-line treatment was considered to be followed from the end of second-line treatment until the patient died (or until the end of the ten-year modelling period). Third-line treatment was with pemetrexed and cisplatin in all cases.

#### Clinical inputs

The principal clinical inputs for the model were derived from the ALEX trial [13] and the outcomes of this trial used in building the model are listed in Table 1.

**Table 1:** Outcomes of the ALEX trial used to set clinical inputs for the model.

Variable	Alectinib	Crizotinib
Overall survival rate at study end (% [95% CI])	77% (n = 117)	73.5% (n = 111)
Progression-free survival (median [95% CI]; months)	25.7 [19.9 –NE]	10.4 [7.7–14.6]
Assessed by IRC		
Treatment duration (median [range]; months)	18.6 [0.5–29.0]	17.6 [0.3–27.0]
Cumulative incidence of brain metastases (% [95% CI])	9.4% [5.4–14.7]	41.4% [33.2–49.4]
Overall incidence of adverse events		
Acute kidney failure	2.0%	0.0%
Aspartate aminotransferase elevation	3.9%	13.9%
Anaemia	2.0%	0.0%
Alanine aminotransferase elevation	5.3%	9.3%
Bilirubinaemia increased	2.0%	0.0%
Diarrhoea	0.0%	2.0%
QT interval prolongation	0.0%	3.3%
Nausea	0.0%	2.0%
Neutropenia	0.0%	2.6%
Infections	1.3%	2.0%
Vomiting	0.0%	2.0%

CI: confidence interval; NE: not estimable.

For OS and PFS over the period corresponding to the length of the ALEX trial, the areas under the curve (AUC) of the Kaplan-Meier functions observed in the trial for alectinib and crizotinib were applied in the model. The proportion of patients in the PPS state at each time interval was determined by subtraction of the AUC for PFS from the AUC for OS.

Beyond the length of the trial, data were extrapolated following the method recommended by the National Institute for Health and Care Excellence (NICE) [18], in the absence of French recommendation. In a first-step, the proportional hazard hypothesis was evaluated by log-cumulative transformation. Since this hypothesis was not confirmed, different mathematical functions were tested for quality of fit to the survival data in the ALEX trial using Akaike and Bayesian information criteria in order to select the most appropriate function for each outcome measure. For PFS and total treatment duration, exponential functions were chosen for alectinib and crizotinib long-term extrapolations. For crizotinib OS, a gamma function was chosen as this matched closely the real five-year survival data observed in the PROFILE 1014 clinical trial [19], which is the best current source of data on real-life practice with the use of ALK inhibitors in real-life clinical practice. For alectinib OS, beyond the length of ALEX trial, transition probabilities were directly reported from crizotinib OS Gamma extrapolation in order to hypothesize no incremental efficacy of alectinib compared to crizotinib beyond the length of ALEX trial.

With regard to BM, it was assumed for the first cycle of the model that 25% of patients would already have BM, since between 15% and 35% of patients with lung cancer have BM at diagnosis [20]. Early incidence of BM over the lifetime of the model was taken from the ALEX trial [13] and subsequent incidence extrapolated using an exponential function for alectinib and crizotinib.

Only AEs classed as Grade III or IV severity, and reported in >2% of patients in or the other of the two treatment groups in the ALEX trial were considered. Occurrence of these AEs was assumed to be linear, and the rate determined as the total incidence rate in the ALEX trials divided by the mean treatment duration. These rates have been transformed to obtain weekly probabilities, applied to each cycle. To model all AEs with Grade III or IV from the ALEX trial, a weighting of the incidence frequencies of identified AEs has been applied.

#### Utility inputs

Evolution of quality of life over the lifetime of the model was taken into account as quality-of-life adjusted life years, determined as the product of the time spent in each health state and the quality of life associated with each health state expressed as a utility measure. The utility measure could take a value between 1 (perfect health) and 0 death. Utility values for the different health states in patients with NSCLC were obtained using the three level EuroQoL five-dimension questionnaire (EQ-5D-3L) (EuroQol Research Foundation) [21] with the application of French tariffs [22]. Data from a previously published study [23] were used to assign a utility value to PPS during third-line treatment and EQ-5D-3L data collected directly in the ALEX trial (unpublished data) were used for PFS and PPS during second-line treatment. The utility values applied in each health state are presented in Table 2.

**Table 2:** Variables evaluated in the deterministic sensitivity analysis.

Variable	Base case	Lower limit	Upper limit	Justification
Age	55 years	44.04 years	66.06 years	± 20%
Utility in PFS	0.831	0.828	0.835	95% CI
Utility progression 2^nd^ line	0.743	0.726	0.760	95% CI
Utility progression 3^rd^ line	0.586	0.469	0.631	95% CI
Patients without BM at entry	75%	65%	85%	Published range [20]
Discount rate	4%	1.5%	6%	Range used in EU countries
Administration cost chemotherapy	€ 419.57	€ 335.66	€ 503.48	± 20%
Monitoring cost for alectinib	€ 23.76	€ 19.06	€ 28.46	± 20%
Monitoring cost for crizotinib	€ 23.76	€ 19.06	€ 28.46	± 20%
Monitoring cost 2^nd^ line PP	€ 23.68	€ 18.94	€ 28.42	± 20%
Monitoring cost 3^rd^ line PP	€ 24.40	€ 19.52	€ 29.28	± 20%
Treatment duration 2^nd^ line	50.1 weeks	40.1 weeks	60.1 weeks	± 20%
Management cost for AE alectinib	€ 10.88	€ 8.70	€ 13.06	± 20%
Management cost for AE crizotinib	€ 18.97	€ 15.18	€ 22.77	± 20%
Management cost for BM/week	€ 114.30	€ 91.44	€ 137.16	± 20%

AE: adverse events; BM: brain metastases; CI: confidence intervals; EU: European Union; PP: post progression; PPS: post progression survival.

These utilities were adjusted by disutility increments associated with the occurrence of adverse events (Table 3). When available, published data [24, 25] were used to assign these disutilities. If no information was available, the mean value of the published disutility increments was assigned. No disutility adjustment was made for the presence of BM, since no information is available on the impact of BM on quality of life measured with the EQ-5D-3L.

**Table 3:** Disutilities attributed to adverse events.

Adverse event	Incremental disutility	Source
Acute renal failure	-0.061	Mean increment assigned
Alanine aminotransferase elevation	-0.061	Mean increment assigned
Anaemia	-0.073	Westwood et al. [25]
Aspartate aminotransferase elevation	-0.061	Mean increment assigned
Diarrhoea	-0.047	Nafees et al. [24]
Increased bilirubinaemia	-0.061	Mean increment assigned
Infection (*eg* pneumonia or pneumonitis)	-0.061	Mean increment assigned
Nausea	-0.047	Nafees et al. [24]
Neutropenia	-0.090	Nafees et al. [24]
QT interval increase	-0.061	Mean increment assigned
Vomiting	-0.048	Nafees et al. [24]

#### Cost inputs

All costs were expressed in 2017 Euros. Acquisition costs for medications were based on the recommended retail price (and dispensing fees) and are listed in Table 2. Costs of intravenous administration of pemetrexed and cisplatin corresponded to the cost of day hospitalisation for chemotherapy according to the production costs based on the French National cost study (ENCC) (Table 2). Details on unit costs and administration regimens used to calculate these treatment costs are provided in the S1 Table on line. Monitoring costs were assigned on the basis of recommended monitoring in the prescribing information for each treatment and using social security tariffs. Details on frequency of monitoring and costs assigned are provided in the Table 2.

Concerning follow-up in community medicine, there is no consensus on the optimal monitoring programme for patients with NSCLC in current French practice guidelines [6]. Advice from the scientific committee proposed a hypothetically standard follow up in terms of frequency of consultations, medical acts, and biological tests. Unit costs of consultations were determined from data collected by the NHI on the total number of consultations by medical speciality in 2014 and the total fees paid to all physicians in France for these consultations. Unit costs of medical acts and biological tests were determined from NHI tariffs and are presented in S2 Table.

The cost of management of BM was identified from a recent hospital costing analysis, which determined the incremental hospitalisation cost associated with BM in patients managed for NSCLC [26]. This covered all outpatient or residential visits to hospital for NSCLC. Costs were attributed from French national tariffs for medical acts applicable in France from 2013 to 2015, updated to 2017 Euros. The standard national tariff was applied to each hospitalisation based on the DRG code attributed in the French hospitalisation database. These standard tariffs include medical and related procedures, nursing care, treatments (except specific expensive drugs), food and accommodation, and investment costs for hospitalised patients. The extra cost was €461/month. To this cost was added the cost of consulting community neuro-oncologists. On the basis of advice from the scientific committee, a consultation rate of 28% of patients (15% of patients during 1^st^ line treatment; 30% during 2^nd^ and 40% during 3^rd^ line) consulting every ten weeks was retained. The consultation was costed on the basis of French national tariffs at € 35.35. A mean monthly community cost of € 4.31 was thus added to the hospitalisation cost to generate a total incremental cost of €495.31 attributable to BM.

The cost of management of Grade III/IV AEs was determined from published reports of these costs in the French setting, one in patients with advanced NSCLC treated in first line [27] and the second in patients with metastatic melanoma [28]. The costs covered included hospitalisations, consultations, treatments and laboratory tests and were valued from the NHI perspective. The melanoma study covered both hospital costs and community costs, whereas the advanced NSCLC study only covered hospital costs. For AEs which were not covered in these two publications, the cost of hospitalisation applied used the production costs based on the French National cost study (ENCC) [29] was used. The advanced NSCLC study provided the proportion of patients hospitalised for each AE. For AEs not described in this publication, it was assumed that 25% of patients with AEs of interest would be hospitalised, this being the mean hospitalisation rate in the advanced NSCL study [27], with the exception of acute kidney failure, for which it was assumed that all patients would require hospitalisation. These unit costs were updated to 2017 Euros, and a transportation cost of € 79.95 for the return visit to the hospital was added. The costs used in the model for each AE are provided in S3 Table.

According to official list prices for alectinib and crizotinib, monthly acquisition costs were € 4,993.63 and € 4,473.07 for alectinib and crizotinib respectively.

### Model outputs

Outcome was modelled as life years gained and progression-free life years gained. From this information, QALYs could be calculated by applying the appropriate utility values. Costs incurred over the model life time were consolidated by type of expense and overall. Cost-effectiveness was determined as the ICER expressed as cost per QALY gained.

### Sensitivity analysis

Two forms of sensitivity analysis were performed, namely a deterministic approach and a probabilistic approach. In the deterministic approach, key model inputs were varied within a range corresponding to the extreme values reported in the source publication. If these were not available, or not pertinent (for example cost of chemotherapy administration), an arbitrary range of ± 20% was applied. The variables used in the deterministic sensitivity analysis and the range of values applied are listed in Table 2. Acquisition costs for treatments were not included to sensitivity analyses since both treatment have a recent official list price.

The probabilistic sensitivity analysis evaluated uncertainty in the value of certain of the input variables according to their distributions (Table 4). Monte Carlo simulations were performed with 1000 iterations, and the results expressed as the cost-effectiveness acceptability curve.

**Table 4:** Variables evaluated in the probabilistic sensitivity analysis.

Variable	Distribution	Distribution parameters
Health state utilities	Gamma	Mean and standard deviation
Costs Treatment administration Treatment monitoring Management of brain metastases	Log-normal	Mean and standard deviation
Progression free survival	Multivariate normal	Cholesky decomposition
Overall survival	Multivariate normal	Cholesky decomposition
Treatment duration	Multivariate normal	Cholesky decomposition

Finally, a scenario sensitivity analysis was performed in which the treatment pathway was more representative of reflect real-life practice according to the scientific committee of the study. For patients in the first-line alectinib cohort, second line therapy was ceritinib in 50% of the cohort and pemetrexed with cisplatin in 50%. In the first-line crizotinib cohort, second line therapy was ceritinib in 20% of cases and alectinib in 80%. Third-line therapy was pemetrexed with cisplatin in all patients for both groups.

## Results

### Clinical outcomes

The progression of patients through the three health states is illustrated in Table 5. The probability of being in the PFS health state remained higher for longer with alectinib compared to crizotinib. Projected mean overall survival was 4.62 years for alectinib and 4.18 years for crizotinib. Projected mean PFS was 30.30 months for alectinib and 16.13 months for crizotinib.

**Table 5:** Patient flow through the health states of the model (10 year horizon).

	Crizotinib			Alectinib		
	PFS	PPS	Death	PFS	PPS	Death
Year 1	46.12%	36.34%	17.55%	66.53%	17.76%	15.71%
Year 2	23.28%	41.75%	34.67%	49.81%	23.15%	27.04%
Year 3	11.66%	42.59%	45.75%	35.27%	27.58%	37.15%
Year 4	5.76%	40.52%	53.71%	24.98%	28.65%	46.37%
Year 5	2.85%	37.44%	59.71%	17.69%	28.99%	53.32%
Year 6	1.39%	34.13%	64.48%	12.44%	28.71%	58.85%
Year 7	0.69%	31.08%	68.24%	8.81%	27.99%	63.20%
Year 8	0.34%	28.34%	71.32%	6.24%	26.99%	66.77%
Year 9	0.17%	25.94%	73.89%	4.42%	25.83%	69.75%
Year 10	0.08%	23.84%	76.08%	3.13%	24.59%	72.28%

PFS: progression-free survival; PPS: post-progression survival.

### Quality-adjusted life years

**Fig 1:**
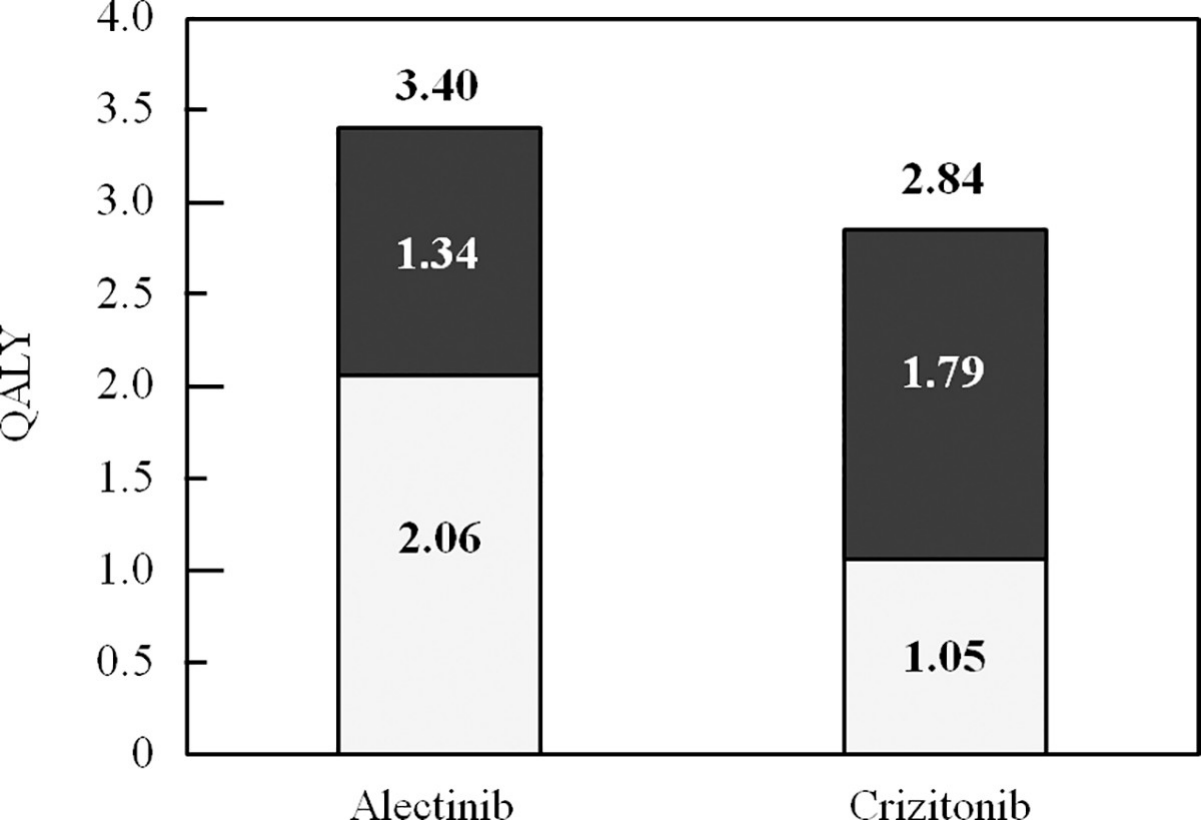
Quality-adjusted life years by health states. Light columns: progression-free survival; dark columns: post-progression.

The total number of quality-adjusted life years projected for alectinib was 3.40, compared to 2.84 for crizotinib, an increase of 20%. This overall benefit was accounted for by a doubling of the quality-adjusted life years in the PFS health state (Fig 1).

### Cost outcomes

The projected total cost of treatment over the ten-year lifetime of the model was € 246,022 for alectinib and € 195,486 for crizotinib, a difference of € 50,535. The majority of this extra cost was related to treatment acquisition costs and management during the PFS health state (Table 6). On the other hand, alectinib was associated with lower costs related to brain metastases and to management during the PPS health state.

**Table 6:** Total costs–base-case scenario.

	Alectinib	Crizotinib	Difference
Treatment acquisition costs	€ 146,599	€ 72,888	€ 73,711
Treatment administration and monitoring costs	€ 630	€ 230	€ 400
Adverse event management costs	€ 1,384	€ 1,340	€ 44
Management during progression-free survival	€ 3,130	€ 1,667	€ 1,463
Management during post-progression survival	€ 82 004	€ 104,051	**-€ 22,047**
Costs associated with brain metastases	€ 12,275	€ 15,312	**-€ 3 037**
**TOTAL**	**€ 246,022**	**€ 195,486**	**€ 50,535**

### Cost-effectiveness

The incremental cost per life year gained was 115,334 €/year and the ICER per QALY gained was 90,232 €/QALY.

### Sensitivity analysis

**Fig 2:**
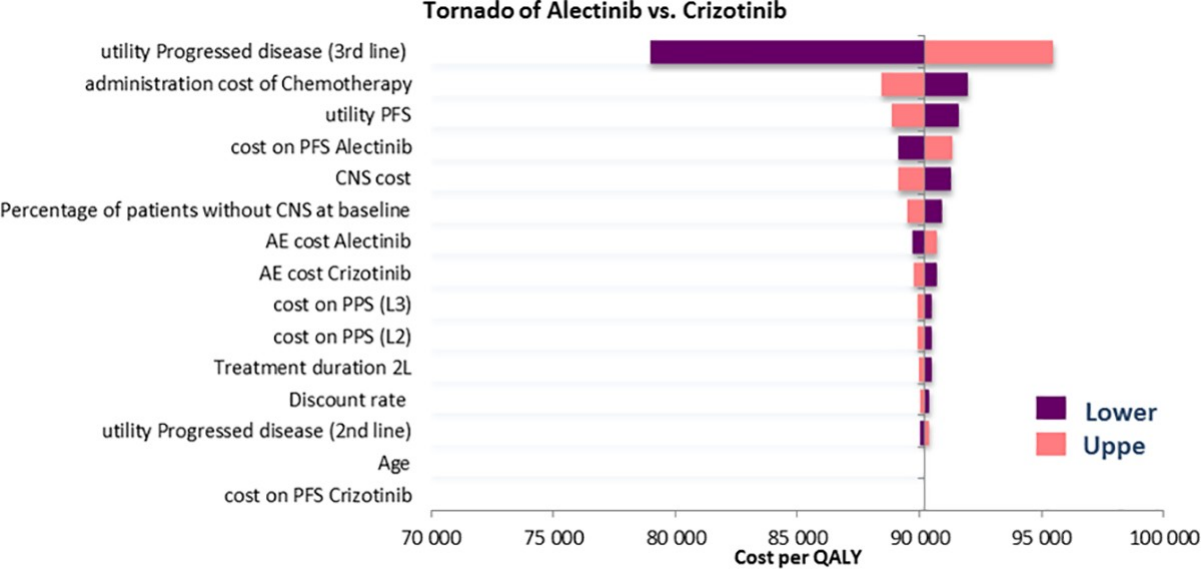
Deterministic sensitivity analysis.

The results of the deterministic sensitivity analysis are presented in the form of a tornado plot in Fig 2. The variables that had the most impact on the ICER were the acquisition costs of alectinib and of crizotinib. Variation of any of the other parameters led to changes in the ICER which remained within the range 79,000 to 95,500 €/QALY, corresponding to a change of <15%.

**Fig 3:**
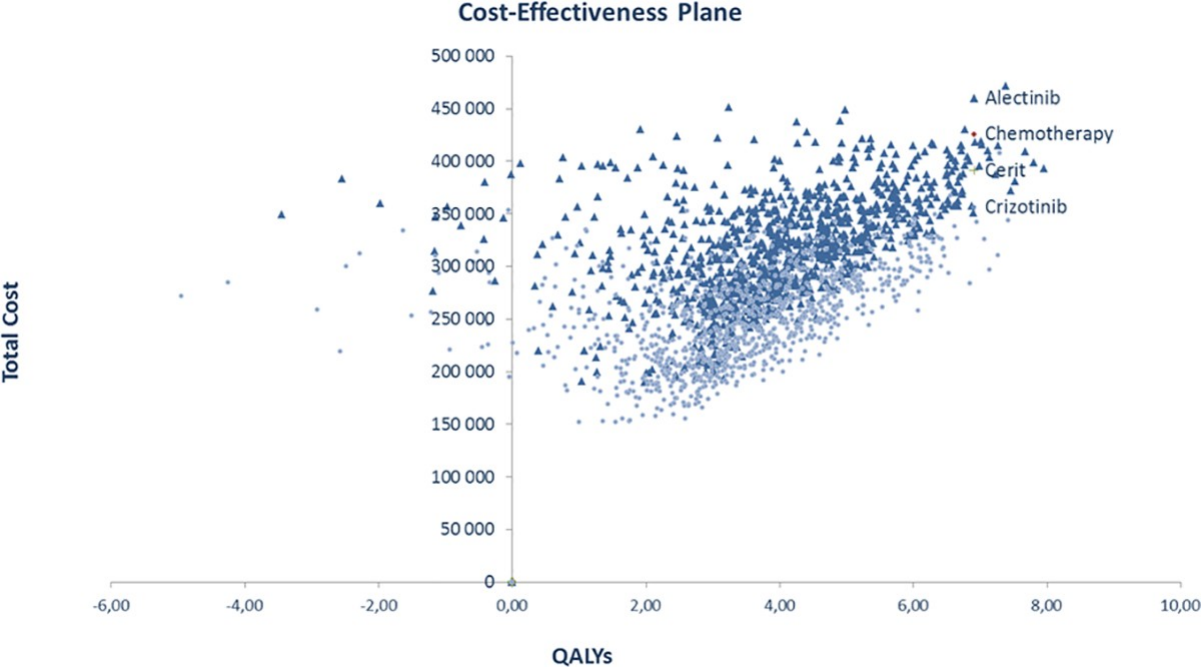
Probability sensitivity analysis.

**Fig 4:**
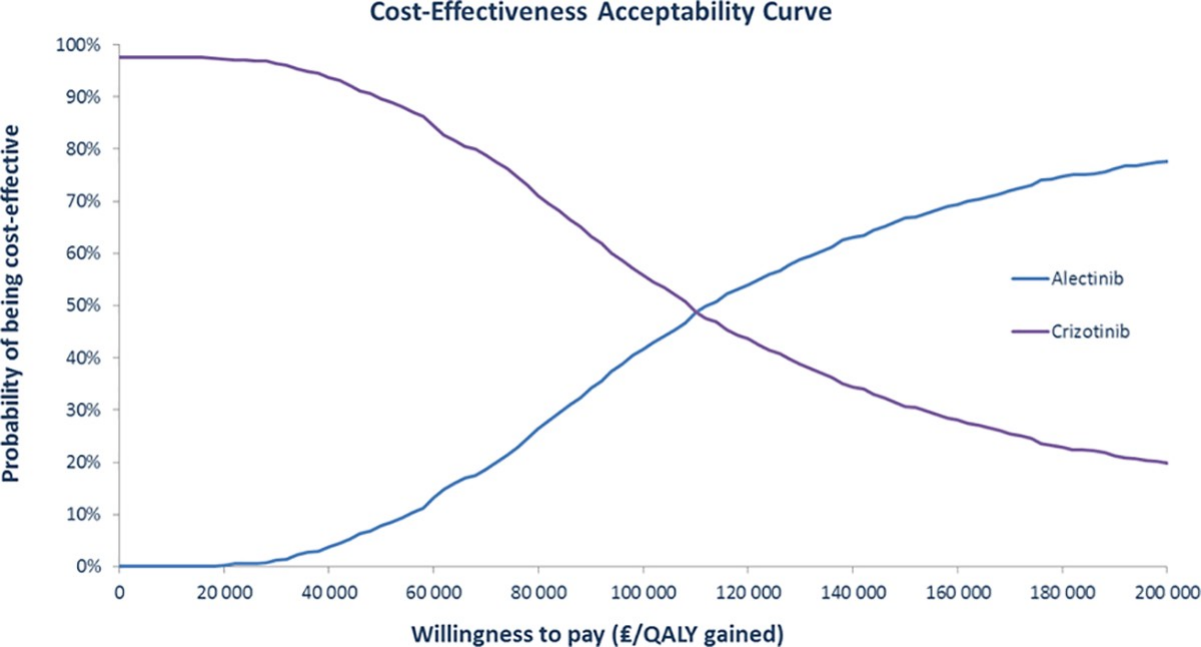
Cost effectiveness acceptability curve.

In the probabilistic sensitivity analysis, the median ICER obtained through 1,000 Monte Carlo simulations was 116,831 €/QALY (Fig 3). The cost-effectiveness acceptability curve is presented in Fig 4. Alectinib would be cost-effective in 50% of cases at a willingness-to-pay threshold of € 110,000 and in 70% of cases at a willingness-to-pay threshold of € 162,000.

The scenario sensitivity analysis modelled the scenario in which patients taking alectinib in first line could be moved to pemetrexed and cisplatin in second line. In this case, the ICER was reduced to € 43,466 €/QALY.

## Discussion

This modelling study was performed to determine the cost-effectiveness of alectinib for the first-line treatment of ALK+ NSCLC in the French setting from a collective perspective. Treatment with alectinib led to a longer projected mean OS duration (4.6 years versus 4.2 years) and mean PFS (30 versus 15 months) against crizotinib. This difference translated into QALYs of 3.40 for alectinib compared to 2.84 for crizotinib. Total costs were € 246,022 for alectinib and € 195,486 for crizotinib, and the ICER with respect to crizotinib was 90,232 €/QALY.

Cost effectiveness analysis was based on the recommended French health authorities guidelines [30] and relevant inputs for the French setting were used where available. In particular, attention was paid to include in the model as many possible variables that might affect clinical outcome and cost and to explore the weight of these variables in sensitivity analysis. Although most of the input variables could be documented from relevant sources, information on community care and on the treatment pathways followed by patients starting first-line treatment with ALK inhibitors was not available and these variables were adjudicated by the scientific committee. The principal clinical inputs were derived from the ALEX trial [13] and the output measures are thus critically dependent on the reliability of the ALEX data. However, the findings of the latter trial are consistent with those of a similar study performed in Japan (J-ALEX) [14].

When choices had to be made for inputs into the baseline model, conservative options were taken in order to minimise bias. This was notably the case for the extrapolation of PFS curves beyond the horizon of the ALEX Trial (choice of an exponential function for alectinib), the definition of the treatment pathway for second- and third- line treatment (choice of a second ALK inhibitor in second line for all patients), the decision not to consider any differential OS efficacy between treatments beyond the end of the observation period of the ALEX trial, the decision not to adjust utility values for disutility related to brain metastases adjustment and the decision not to include palliative care costs or expensive immunotherapy as last-chance therapy. For the extrapolation of OS, it should be noted that even if this extrapolation is less conservative than that selected by the French Health Authorities for evaluating the cost-effectiveness of alectinib, it yields a better match to the actual survival data observed in the PROFILE 1014 trial for crizotinib [19]. In our model, long-term OS with alectinib was hypothesised to be equivalent to that of crizotinib, following the conservative approach recommended by NICE [18], and the same extrapolation was this used for the two agents. Only Grade III or IV severity adverse events were considered as these are expected to generate the most significant costs. However, it is possible that certain Grade II severity adverse events may be clinically relevant and affect hospitalisation, morbidity rates and costs. For example, persistent Grade 2 diarrhoea is a very frequent reason for hospital admissions. Not including such events may affect the outcomes of the model if their incidence is high and differs significantly between the two treatment pathways modelled.

A specificity of the present modelling study was to include the impact on clinical and cost outcomes of the occurrence or progression of brain metastases using data from the ALEX study [13] to model the evolution of brain metastases over time and data from a recent hospital database analysis [26] to model costs. This is of particular relevance given that management of these metastases in NSCLC is a major unmet medical need [11], where alectinib can bring an important clinical benefit [12]. The modelling study indicates that use of alectinib in first-line treatment can result in significant cost savings compared to crizotinib in the context of the French health care system. In addition, it is possible that the cost of brain metastases included in the model are underestimated, since, as pointed out in the source publication [26], only hospital costs and consultation by neuro-oncologist are considered and costs incurred in the community or in nursing homes, which may be considerable [31], are not taken into account.

The comparator used in this study was crizotinib, at the time the only ALK+ inhibitor approved for first-line treatment of advanced ALK+ NSCLC in France and the recommended first-line treatment for this form of lung cancer in European guidelines [5]. Since the present study was initiated, ceritinib has also been licensed for first-line treatment of advanced ALK+ NSCLC (December 2017). However, no randomised clinical trials have been performed which directly compared ceritinib and alectinib which could be used as the basis for a modelling study of the relative cost-effectiveness of these two agents.

The major source of extra cost associated with first-line alectinib treatment compared to crizotinib was associated with treatment acquisition costs in the French public sector. This extra cost is not due to a difference in price between the two ALK inhibitors, indeed crizotinib is marginally cheaper than alectinib. The differences arises because patients treated with alectinib as first line remain on first- and second- line ALK inhibitor treatment for longer before moving to third-line chemotherapy (pemetrexed and cisplatin) which is significantly cheaper. Treatment acquisition cost thus depends critically on the treatment pathway through the different lines of treatment. In the scenario sensitivity analysis, in which a proportion of patients treated with alectinib would be switched to chemotherapy in second-line, considered to represent real-world clinical practice more faithfully, the ICER decreased dramatically. In line with this, the deterministic sensitivity analysis indicated that acquisition costs of ALK inhibitors were the major variables affecting the precision of the ICER estimate.

The results obtained here for the French healthcare setting can be compared with those of a cost-effectiveness study comparing alectinib to crizotinib in the US context. This evaluation differed from the present one in that it used a Markov model rather than a partition survival model, and the extrapolation for crizotinib and alectinib OS was modelled on independent exponential functions which was a more optimistic approach for alectinib than that used in the present model. However in both models, the clinical inputs for this study came from the ALEX trial. Although comparison between different models should be made with caution, clinical outcomes were somewhat more favourable than in the present model, costs were similar and the ICER/QALY was lower. A cost-effectiveness study has also been performed in the Greek setting, currently only published in abstract form [32], which reported a higher ICER, driven principally by a considerably lower acquisition cost for crizotinib.

A health technology appraisal of alectinib has recently been performed by NICE [33], which included a cost-effectiveness evaluation based on a modelling study performed by the manufacturer. This used a similar partition survival model to this one, although it differed by the presence of four health states (PFS, PPS with brain progression, PPS without brain progression and death), and like the US cost-effectiveness study, modelled long-term OS using an exponential function. The NICE committee concluded that alectinib was cost-effective in its approved indication. The present French model differs from the previous US and English models in that, given the uncertainty regarding long term benefit, no differential efficacy was assumed beyond the 29-month observation period of the ALEX trial. The US and English models both assumed that benefits arising from a superior efficacy of alectinib compared to crizotinib would accrue until the end on the model lifetime (lifetime in the US model and 15 years in the English one).

Unlike, for example NICE in England and Wales, the Economic and Public Health Evaluation Committee in France does not apply an explicit willingness to pay threshold for evaluating the cost-effectiveness of new medications, and this is judged on a case-by-case basis. For example, recent approvals of oncology treatments have concerned treatments with ICERs that have ranged from € 26,088/QALY (enzalutamide) to € 266,219/QALY (palbociclib). In the specific case of NSCLC, the ICER for nivolumab was estimated to be €140 106/QALY for second-line treatment of squamous cell NSCLC and € 145,405/QALY for second-line treatment of non-squamous cell NSCLC [34, 35]. In its recent evaluation of alectinib, the French health authority stated that, in light of the superior efficacy and safety of alectinib compared to crizotinib demonstrated in the ALEX trial, alectinib should be the treatment of choice in the first line treatment of ALK+ lung cancer [36].

In conclusion, this cost-effectiveness study indicates that first-line treatment of ALK+ NSCLC with alectinib provides superior clinical outcomes to crizotinib and is cost-effective for the French Health care system. The higher cost of alectinib is primarily attributable to the extended time that patients spend free of progression before being placed on standard chemotherapy. The model used illustrates the interest of including treatment-specific outcomes, in this case evolution of CNS disease, in cost-effectiveness evaluations in order to capture the costs and utilities that differentiate alternative treatment options.

## Supporting information

S1 Table Determination of treatment costs. ^a^including any pharmacy costs for delivery (€1.02 for community pharmacies: alectinib, crizotinib and ceritinib).
^b^It was assumed that each patient would go to and from the hospital in a medicalised vehicle, the cost of which is reimbursed by social security. The French Court of Audit reported that 50.1 million such transports were reimbursed in 2010, at a total cost of € 1 900 milion. This represents a unit cost of € 37.92 per trip, or €79.95 for a return trip (adjusted for inflation).
^c^As some latitude is envisaged in the prescribing information, the values given represent the monitoring rate chosen for the modelling study.
*bid*: twice a day.
^d^In addition, a fixed charge of € 9.18 is levied for each monitoring visit involving blood sampling, regardless of the nature of the test performed.
(DOCX)

S2 Table Determination of costs of consultations and procedures. Source: Mean physician consultation tariffs are published annually by the French Health Insurance (https://www.ameli.fr/l-assurance-maladie/statistiques-et-publications/donnees-statistiques/professionnels-de-sante-liberaux/honoraires/honoraires-totaux-et-moyens.php), as are unit costs for medical procedures (Classification Commune des Actes Médicaux: https://www.ameli.fr/accueil-de-la-ccam/index.php).
^a^Procedure code in the *Classification Commune des Actes Médicaux*.
(DOCX)

S3 Table Determination of costs of management of adverse events. (DOCX)
