# Supplementary material for: Cost-effectiveness of alectinib compared to crizotinib for the treatment of first-line ALK+ advanced non-small-cell lung cancer in France
Source: PLoS One. 2020 Jan 16;15(1):e0226196. doi: 10.1371/journal.pone.0226196 (PMC6964893; doi:10.1371/journal.pone.0226196)
Supplement: S1 Table — aincluding any pharmacy costs for delivery (€1.02 for community pharmacies: alectinib, crizotinib and ceritinib). bIt was assumed that each patient would go to and from the hospital in a medicalised vehicle, the cost of which is reimbursed by social security. The French Court of Audit reported that 50.1 million such transports were reimbursed in 2010, at a total cost of € 1 900 milion. This represents a unit cost of € 37.92 per trip, or €79.95 for a return trip (adjusted for inflation). cAs some latitude is envisaged in the prescribing information, the values given represent the monitoring rate chosen for the modelling study. bid: twice a day. dIn addition, a fixed charge of € 9.18 is levied for each monitoring visit involving blood sampling, regardless of the nature of the test performed. (DOCX) [file pone.0226196.s001.docx]

S1 Table.

| **ACQUISITION COSTS** | | | | |
| --- | --- | --- | --- | --- |
| **Treatment** | **Dose regimen/ packaging** | **Unit (pack) costs** | **Weekly cost**^a^ | **Source** |
| Alectinib | 4 × 150 mg capsules *bid* Blister packs of 56 × 150 capsules | € 4,592.71 | € 1,152.38 | Listed retail price  Dispensing fees |
| Crizotinib | 1 × 250 mg capsule *bid* Blister packs of 60 × 250 capsules | € 4,407.763 | € 1,032.25 | Listed retail price  Dispensing fees |
| Ceritinib | 5 × 150 mg capsules/day Blister packs of 150 × 150 mg capsules | € 4,911.01 | € 1,150.08 | Listed retail price  Dispensing fees |
| Pemetrexed | 500 mg/m² infusion on Day 1 ; 21 day cycle. Vials of 500 mg or 100 mg | € 602.339 | € 1,043.01 | Extra-DRG funding (“liste en sus”)  French National cost study (ENCC) |
| Cisplatin | 75 mg/m² infusion on Day 1 ; 21 day cycle. Vials of 50 ml |  |  |  |
| **ADMINISTRATION COSTS** | | | | |
| **Treatment** | **Dose regimen** | **Administration cost / month** | **Transport**^b^ | **Source** |
| Pemetrexed + cisplatin | As above | € 304.25 | € 79.95 | Official hospital tariffs |
| **MONITORING COSTS** | | | | |
| **Treatment** | **Type of monitoring** | **Monitoring rate**^c^ | **Cost of test**^d^ | **Source** |
| Alectinib | Liver function | Every 2 weeks for 1^st^ month, then monthly | € 2.97 | Social security tariffs |
|  | Fasting blood glucose | Monthly | € 7.56 |  |
| Crizotinib | Liver function | Weekly for 2 months, then twice monthly | € 2.97 |  |
| Ceritinib | Liver function | Every 2 weeks for 1^st^ month, then monthly | € 2.97 |  |
|  | Fasting blood glucose | Monthly | € 7.56 |  |
| Pemetrexed | Full blood count | Every 3 weeks (before treatment) | € 7.83 |  |
|  | Liver function |  | € 2.97 |  |
|  | Renal function |  | € 8.10 |  |
| Cisplatin | Liver function | Every 3 weeks (before treatment) | € 2.97 |  |
|  | Renal function |  | € 8.10 |  |
|  | Haematopoietic function |  | € 7.83 |  |
|  | Serum electrolytes |  | € 7.29 |  |
