# Supplementary material for: Cost-effectiveness of alectinib compared to crizotinib for the treatment of first-line ALK+ advanced non-small-cell lung cancer in France
Source: PLoS One. 2020 Jan 16;15(1):e0226196. doi: 10.1371/journal.pone.0226196 (PMC6964893; doi:10.1371/journal.pone.0226196)
Supplement: S2 Table — Source: Mean physician consultation tariffs are published annually by the French Health Insurance (https://www.ameli.fr/l-assurance-maladie/statistiques-et-publications/donnees-statistiques/professionnels-de-sante-liberaux/honoraires/honoraires-totaux-et-moyens.php), as are unit costs for medical procedures (Classification Commune des Actes Médicaux: https://www.ameli.fr/accueil-de-la-ccam/index.php). aProcedure code in the Classification Commune des Actes Médicaux. (DOCX) [file pone.0226196.s002.docx]

S2 Table.

|  | Line 1 | | | Line 2 | | | Line 3 | | |
| --- | --- | --- | --- | --- | --- | --- | --- | --- | --- |
|  | Frequency /week | Cost per resource | Cost /week | Frequency /week | Cost per resource | Cost /week | Frequency /week | Cost per resource | Cost /week |
| Neuro-oncologist | Included in BM cost | | | | | | | | |
| Oncologist | 0.23 | 35.35 | 8.13 | 0.23 | 35.35 | 8.13 | 0.23 | 35.35 | 8.13 |
| GP | 0.05 | 31.19 | 1.43 | 0.07 | 31.19 | 2.15 | 0.09 | 31.19 | 2.87 |
| Radiologist | 0.09 | 51.86 | 4.77 | 0.08 | 51.86 | 3.97 | 0.08 | 51.86 | 3.97 |
| CT scan (ZBQH001)^a^ | 0.10 | 25.27 | 2.53 | 0.10 | 25.27 | 2.53 | 0.10 | 25.27 | 2.53 |
| MRI (ECQJ001)^a^ | 0.10 | 69.00 | 6.90 | 0.10 | 69.00 | 6.90 | 0.10 | 69.00 | 6.90 |
| **Total** | **€ 23.76** | | | **€ 23.68** | | | **€ 24.40** | | |
