# Supplementary material for: Cost-effectiveness of alectinib compared to crizotinib for the treatment of first-line ALK+ advanced non-small-cell lung cancer in France
Source: PLoS One. 2020 Jan 16;15(1):e0226196. doi: 10.1371/journal.pone.0226196 (PMC6964893; doi:10.1371/journal.pone.0226196)
Supplement: S3 Table — (DOCX) [file pone.0226196.s003.docx]

S3 Table.

| **Adverse event** | **Patients hospitalised (%)** | **Hospitalisation cost** | **Ambulatory cost** | **Total cost including transportation** | **Source** |
| --- | --- | --- | --- | --- | --- |
| Acute renal failure | 100% | € 4,535.27 |  | € 4,615.22 | GRD tariff |
| AST elevation | 25% | € 6,913 | € 28 | € 1,733.93 | Melanoma study [28] |
| Anaemia | 42% | € 5,258 | | € 4,976.58 | NSCLC study [27] |
| ALT elevation | 25% | € 6,913 | € 28 | € 1,733.93 | Melanoma study [28] |
| Total bilirubin elevated | 25% | € 3,444.07 |  | € 881.01 | GRD tariff |
| Diarrhoea | 32% | € 2,632 | | € 2,499.90 | NSCLC study [27] |
| Prolonged QT interval | 25% | € 2,468.50 |  | € 637.11 | GRD tariff |
| Nausea | 50% | € 1,876  € 85 | | € 1,915.98 | NSCLC study [27] |
| Neutropenia | 2% |  |  | € 86.60 | NSCLC study [27] |
| Infection | 25% | € 3,018 | € 67 | € 821.70 | Melanoma study [28] |
| Vomiting | 42% | € 1,923 | | € 1,841.37 | NSCLC study [27] |
